# Reduced subcortical glutamate/glutamine in adults with autism spectrum disorders: a [^1^H]MRS study

**DOI:** 10.1038/tp.2013.53

**Published:** 2013-07-09

**Authors:** J Horder, T Lavender, M A Mendez, R O'Gorman, E Daly, M C Craig, D J Lythgoe, G J Barker, D G Murphy

**Affiliations:** 1 King's College London, Department of Forensic and Neurodevelopmental Sciences, Institute of Psychiatry, London, UK; 2 MR Centre, University Children's Hospital Zurich, Zurich, Switzerland; 3 King's College London, Department of Neuroimaging Sciences, Institute of Psychiatry, London, UK

**Keywords:** autism spectrum disorders, autism, glutamate, glutamine, [^1^H]MRS

## Abstract

Dysfunctional glutamatergic neurotransmission has been implicated in autism spectrum disorder (ASD). However, relatively few studies have directly measured brain glutamate in ASD adults, or related variation in glutamate to clinical phenotype. We therefore set out to investigate brain glutamate levels in adults with an ASD, comparing these to healthy controls and also comparing results between individuals at different points on the spectrum of symptom severity. We recruited 28 adults with ASD and 14 matched healthy controls. Of those with ASD, 15 fulfilled the ‘narrowly' defined criteria for typical autism, whereas 13 met the ‘broader phenotype'. We measured the concentration of the combined glutamate and glutamine signal (Glx), and other important metabolites, using proton magnetic resonance spectroscopy in two brain regions implicated in ASD—the basal ganglia (including the head of caudate and the anterior putamen) and the dorsolateral prefrontal cortex—as well as in a parietal cortex ‘control' region. Individuals with ASD had a significant decrease (*P*<0.001) in concentration of Glx in the basal ganglia, and this was true in both the ‘narrow' and ‘broader' phenotype. Also, within the ASD sample, reduced basal ganglia Glx was significantly correlated with increased impairment in social communication (*P*=0.013). In addition, there was a significant reduction in the concentration of other metabolites such as choline, creatine (Cr) and *N*-acetylaspartate (NAA) in the basal ganglia. In the dorsolateral prefrontal cortex, Cr and NAA were reduced (*P*<0.05), although Glx was not. There were no detectable differences in Glx, or any other metabolite, in the parietal lobe control region. There were no significant between-group differences in age, gender, IQ, voxel composition or data quality. In conclusion, individuals across the spectrum of ASD have regionally specific abnormalities in subcortical glutamatergic neurotransmission that are associated with variation in social development.

## Introduction

Autism spectrum disorder (ASD) is characterized by deficits in social reciprocity, communication impairments, and restricted, repetitive interests and behaviours.^1^ Recent research suggests an approximate prevalence of 0.6–1.5% in the general population.^2^

At present, however, therapeutic options for ASD are limited because the pathophysiology of ASD is unclear, leading to a paucity of treatment targets for the core symptoms. Numerous studies have reported abnormalities in brain anatomy and function of ASD individuals (e.g. see Hallahan *et al.*^3^ and Barttfeld *et al.*^4^), but the underlying molecular basis of these differences is unknown.

There is, however, emerging evidence suggesting that ASD may be associated with abnormalities in excitatory glutamate and inhibitory γ-amino-butyric acid (GABA) neurotransmission.^5^ The balanced interaction between glutamate and GABA transmission is essential for regulating cognition, learning, memory and emotional behaviours. An imbalance between glutamate excitation and GABA inhibition, leading to hyperexcitation, has been linked to ASD.^6, 7, 8^

There is also evidence for an association between ASD and genetic variation in the glutamatergic and GABAergic systems. For example, there are reports of associations between ASD and variants in genes coding for glutamate receptors^9, 10^ and glutamate transporter proteins,^11^ although not in all studies.^12^ Also, recent work on fragile X syndrome, the most common monogenetic syndrome associated with ASD, points to the potential importance of metabotropic glutamate receptors (mGluR1) as possible treatment targets in ASD.^13^

Unfortunately, it is not possible to quantify glutamate and GABA concentrations in post-mortem studies, because they degrade rapidly after death. Progress can be made, however, as *in vivo* proton magnetic resonance spectroscopy ([^1^H]MRS) can be used to quantify a range of neural metabolites, including glutamate and its metabolic product glutamine (Glu+Gln—henceforth abbreviated Glx).

There are six published [^1^H]MRS studies reported on Glx in ASD. Of these, four investigated children, one reported a widespread decrease in cortical Glx^14^ and another reported a nonsignificant reduction in Glx in the left thalamic region.^15^ However, two other studies found no differences in any region studied: one investigated the frontal, temporal and parietal cortex and basal ganglia,^16^ and the other the frontal cortex and basal ganglia.^17^

Only two published [^1^H]MRS studies have measured Glx in adults with ASD. Page *et al.*^18^ reported that adults with ASD had a significantly *higher* concentration of Glx than controls in the right amygdala-hippocampal complex. In contrast, Bernardi *et al.*^19^ found a significantly *lower* Glx in the right anterior cingulate cortex.

These prior investigations suggest that ASD individuals may have differences in brain Glx, but the results are inconsistent. Possible explanations for this mixed picture are that some of these studies investigated relatively small samples, and they examined different age groups and/or brain regions. Also, no study of adults has yet addressed whether any of these putative differences are present across the behavioural spectrum (i.e. in both the ‘core' disorder, and those with the ‘broader phenotype').

This is potentially of importance because while, in the past, autism was generally treated as a ‘categorical' diagnosis, it is now understood to likely cover a spectrum of severity. For example, the biological relatives of people with ASD often show an attenuated ‘broader phenotype' of mild social, cognitive and neurobiological abnormalities.^20^

This clinical (and likely aetiological) heterogeneity has led some to suggest that we refer to ‘the autisms' rather than to ‘autism' and search for final common pathways through which various causative agents may lead to disorder.^21^ However, previous [^1^H]MRS studies of adults with ASD have treated all participants with ASD as a single group (although one study in children did not).^22^ Thus, it is unclear whether putative abnormalities in Glx are present across the spectrum, that is, whether they are a potential common pathway, and/or relate to particular core symptoms.

Hence, in this study, we used [^1^H]MRS to investigate differences in brain glutamate and other metabolites in adults with ASD. We compared controls with ASD people diagnosed with the narrow ‘core' disorder, who scored above cutoff on research diagnostic criteria, and those with a broader phenotype, who only met some of the criteria.

We focused on regions previously implicated in ASD pathology and symptomatology: the basal ganglia and the dorsolateral prefrontal cortex (DLPFC). For example, (1) anatomical and metabolic abnormalities have been reported in both of these interconnected areas in ASD;^23, 24^ (2) the basal ganglia have been linked with social and emotional differences^25^ and compulsive and repetitive behaviours^26^ and (3) the DLPFC has been linked to deficits in executive function^27^ and theory of mind.^28^ Hence, we also correlated [^1^H]MRS measures that differed significantly between groups with scores on the Autism Diagnostic Interview—Revised (ADI-R) interview.

We also included a ‘control' region, in the medial parietal lobe, which has not been linked to ASD and where no differences were seen in a previous [^1^H]MRS study.^18^

## Materials and methods

### Participants

We recruited 42 adult participants: 28 individuals with ASD and 14 healthy controls matched for age, gender and IQ (see Table 1). All participants had an IQ above 65. We recruited only participants who reported being right-handed, to avoid possible lateralization effects given our use of unilateral [^1^H]MRS voxels.

The 28 participants in the ASD group were further divided into two subtypes on the basis of their symptom profile. Fifteen were diagnosed with the ‘narrowly defined phenotype' of autism based on the fact that they met the ADI-R cutoff criteria in all three symptom domains and fulfilled the diagnostic criteria for childhood autism or Asperger's syndrome according to the ICD-10 Research Classification of Mental and Behavioural Disorders^1^ (criteria F84.0 and F84.5, respectively). The other 13 individuals were classified as having the ‘broader phenotype', that is, they did not meet the ADI-R cutoff in one domain (see Table 1), but fulfilled the ICD-10 diagnostic criteria for atypical autism (F84.1).

All individuals with ASD were recruited through London's Maudsley Hospital Behavioural Genetics Clinic, a specialist diagnostic service.

Potential participants were excluded if they had a comorbid psychiatric or medical disorder affecting brain development (e.g. epilepsy or psychosis), a history of head injury, a genetic disorder associated with ASD, for example, tuberous sclerosis or fragile X syndrome, or an IQ below 65. Participants with ASD suffering from anxiety or depressive disorders were not excluded, given the high frequency of these comorbidities in ASD. According to participant self-report, all participants were medication naive at the time of scanning.

All participants provided written informed consent. Ethical approval for this study was provided by South London and Maudsley/Institute of Psychiatry NHS Research Ethics Committee, study reference 1997/087.

### [^1^H]MRS data acquisition

[^1^H]MRS data were acquired on a 1.5 T GE HDx magnetic resonance imaging (MRI) scanner (GE Medical Systems, Milwaukee, WI, USA) equipped with TwinSpeed gradients.

The scanning protocol included a structural MRI scan, namely a three-dimensional fast inversion-recovery-prepared gradient echo acquisition (number of slices=146, slice thickness=1.2 mm, inversion time (TI)=300 ms, repetition time (TR)=11 ms, echo time (TE)=5 ms, field of view=310 mm, flip angle=18°, matrix=256 × 160 over a 310 × 194 mm field of view, giving 1.2 × 1.2 × 1.2 mm^3^ voxels). This structural MRI was used for the localization of the spectroscopy voxels in each participant.

**Figure 1:**
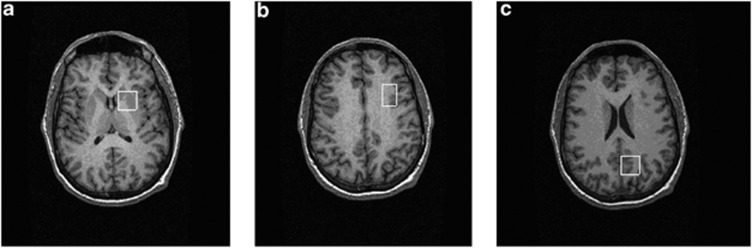
Examples of the location of proton magnetic resonance spectroscopy ([^1^H]MRS) voxels. Three voxels were positioned in (**a**) left basal ganglia (20 × 20 × 15 mm^3^) to include the head of the caudate, putamen and internal capsule, (**b**) left dorsolateral prefrontal cortex (16 × 24 × 20 mm^3^) and (**c**) left medial parietal lobe (20 × 20 × 20 mm^3^).

Single voxel [^1^H]MRS spectra were then acquired, using a point-resolved spectroscopy sequence. Point-resolved spectroscopy parameters were: TR=3000 ms and TE=30 ms. A voxel of interest was positioned in the left basal ganglia (20 × 20 × 15 mm^3^). This voxel included parts of the head of the caudate, the anterior putamen and the internal capsule. Voxel of interests were also positioned in the left DLPFC (16 × 24 × 20 mm^3^) and in the left medial parietal lobe (20 × 20 × 20 mm^3^), using previously described methods.^29^ See Figure 1 for an illustration of the location of the voxels.

### Data processing

[^1^H]MRS spectra were processed using the LCModel software version 6-1-0 (Stephen Provencher Incorporated, Oakville, Canada). LCModel uses a linear combination of model spectra of metabolite solutions *in vitro* to analyse the major resonances of *in vivo* spectra. In this case, a basis set of alanine, aspartate, creatine (Cr), γ-aminobutyric acid (GABA), glutamine, glutamate, glycerophosphocholine, mI, lactate, *N*-acetylaspartate (NAA), *N*-acetyl-aspartylglutamate, scyllo-inositol and taurine, together with a baseline function, were used for the analysis. Each spectrum was reviewed to ensure adequate signal-to-noise ratio, as well as the absence of artefacts. Note that the NAA resonance at 2 p.p.m. contains both NAA and *N*-acetyl-aspartylglutamate; we report here results reflecting the combination of NAA+*N*-acetyl-aspartylglutamate, and use the term NAA for brevity.

### Calculation of absolute metabolite concentrations

Metabolite concentrations for NAA, Cr, Glx and choline (Cho) were calculated, in institutional units, as follows. The raw metabolite estimates (LCModel output) were first corrected by reference to calibration data from a phantom, containing an aqueous solution of known NAA concentration. One phantom [^1^H]MRS spectrum was acquired at the end of each scanning session. The amplitude of the phantom NAA peak was used to derive a correction factor, by which all metabolite values for the scan were multiplied.

Furthermore, partial volume effects (group differences in proportions of gray matter, white matter and cerebrospinal fluid, CSF, in the [^1^H]MRS voxels) are a potential confound in spectroscopy. This could be especially relevant to the present investigation, given previously reported volumetric differences between ASD individuals and controls, for example, in the basal ganglia.^23, 26^

Therefore, to guard against such confounds, we determined the percentage of gray matter, white matter and CSF within each [^1^H]MRS voxel for each participant. We first segmented the T1-weighted structural MRI using an automated procedure, *spm_segment*, part of the Statistical Parametric Mapping software package (SPM2; http://www.fil.ion.ucl.ac.uk/spm/software/spm2/; Wellcome Trust Centre for Neuroimaging, London, UK).

The position of each individual [^1^H]MRS voxel relative to the corresponding structural was determined, using positional coordinates embedded in the raw spectra data files. The % grey, white and CSF composition of each voxel was then calculated automatically using in-house software. Finally, all metabolite concentrations were corrected for the amount of CSF in the voxel—under the assumption that CSF only contains negligible quantities of the metabolites of interest—by multiplying values by an individual correction factor=1/(1−Proportion_CSF_), where Proportion_CSF_ could range from 0 to 1, calculated separately for each voxel from each participant. This was applied after correcting for phantom NAA values (see above).

In summary: Metabolite_corrected_=Metabolite_raw_ × (PhantomNAA_known_/PhantomNAA_observed_) × (1/(1−Proportion_CSF_)).

### Statistical analysis

Age and IQ were compared using one-way analysis of variance (ANOVA) across the three groups (healthy control, broad ASD and narrow ASD).

Differences in mean metabolite concentrations were calculated using a series of one-way ANOVAs, with group as a between-subjects factor. One such ANOVA was performed for each of the four metabolites, in each of the three voxels, a total of 12 ANOVAs. As this procedure involves multiple (12) comparisons, we applied a Bonferroni correction to guard against Type I errors. We report results both before and after this correction.

Planned *post hoc* independent sample *t*-tests were then applied, in metabolites where a significant between-group difference was found on the ANOVA, to evaluate differences between (a) narrow ASD and healthy controls, (b) broader ASD and healthy controls and (c) between the two ASD groups (narrow vs broad).

We examined possible correlations between concentrations of metabolites that differed significantly from controls in these *t*-tests against ADI-R domain scores, across the whole combined ASD group, using Pearson's product–moment correlation coefficients.

All analyses were performed using SPSS 15.0 software (SPSS, Chicago, IL, USA).

## Results

### Demographics

Groups did (Table 1) not differ significantly in age, full-scale IQ, verbal IQ or performance IQ.

### Tissue composition and data quality

Groups did not differ significantly in mean voxel % grey matter, white matter or CSF in any of the three voxels (Table 2). This is unsurprising as, although volumetric differences have been observed in ASD in the basal ganglia^26^ and cortex,^30^ these were of small magnitude, and would not be expected to materially affect composition of hand-placed voxels.

**Figure 2:**
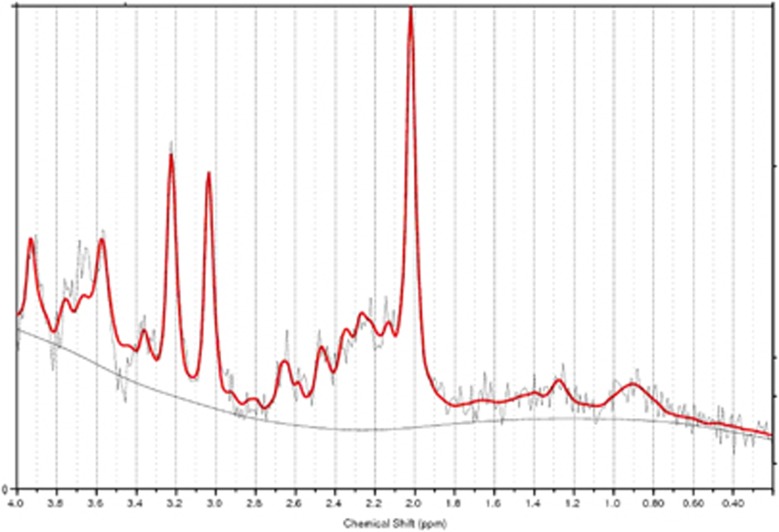
Example of a proton magnetic resonance spectroscopy ([^1^H]MRS) spectrum showing LCModel 6-1-0 fit.

To verify that the quality of the [^1^H]MRS data did not vary between groups, we compared the LCModel 6-1-0 Cramer-Rao Lower Bound estimate standard deviations for each metabolite in each voxel, using a one-way ANOVA across the three groups. This revealed no significant differences (all F(2,42)<2.8, all *P*>0.07.) See Figure 2 for an example of a [^1^H]MRS spectrum after model fitting.

Finally, because of the potential risk of ‘drift' in extended [^1^H]MRS investigations (in which metabolite estimates on the same scanner change over long periods of time), we compared the timings of scans across the three groups, in terms of days after the first scan of the series. This revealed no significant difference (one-way ANOVA F(2,44)=0.739, *P*=0.484). Scan date was also not correlated with the value of any metabolite in any voxel (all Pearson's *r*<0.23, all *P*>0.13).

### Metabolite differences

#### Basal ganglia

**Figure 3:**
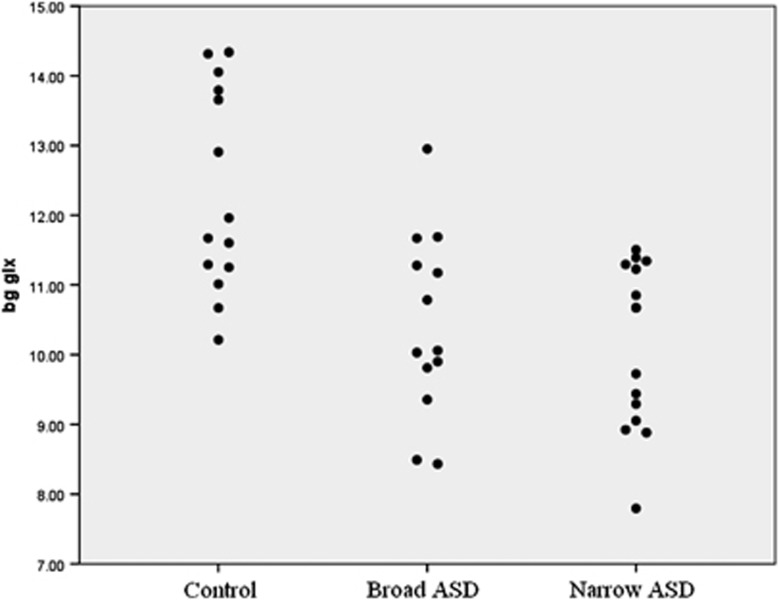
Individual participant data showing basal ganglia glutamate and glutamine (Glx) by group. ASD, autism spectrum disorder.

There was a significant group effect in every metabolite concentration we measured in this voxel (ANOVA) (Table 3). Two of these effects—Cr and Glx—survived conservative Bonferroni correction for multiple comparisons over all metabolites and voxels. *Post hoc* independent sample *t*-tests showed that Glx concentrations were significantly lower in both the ‘restricted' and ‘broader' ASD phenotypes compared with controls (see Figure 3). Both Cr and NAA showed a similar pattern. Cho was also lower in both ASD groups as compared with controls, but this only reached statistical significance in the broader ASD phenotype.

There were no significant differences between the two ASD subgroups in any metabolite concentration.

To establish whether the finding of lower Glx remained significant after controlling for tissue composition and the other metabolite concentrations, we performed a univariate GLM covarying for basal ganglia grey/white matter, Cr, NAA, Cho, age and IQ. The findings remained significant (F=3.530, *P*<0.05).

#### Dorsolateral prefrontal cortex

There was no significant effect of group for Glx or Cho. However, there were significant effects of Cr and NAA, although neither of these differences survived conservative Bonferroni correction. *Post hoc* tests revealed that Cr was significantly lower in both ASD groups relative to healthy controls, whereas NAA was significantly lower only in the ‘narrow' ASD phenotype. However, there were no significant differences between the two ASD subgroups.

#### Parietal region

As predicted, no significant differences were found in any metabolite concentration between any of the groups, even before Bonferroni correction.

### Relationship to behavioural variables

**Figure 4:**
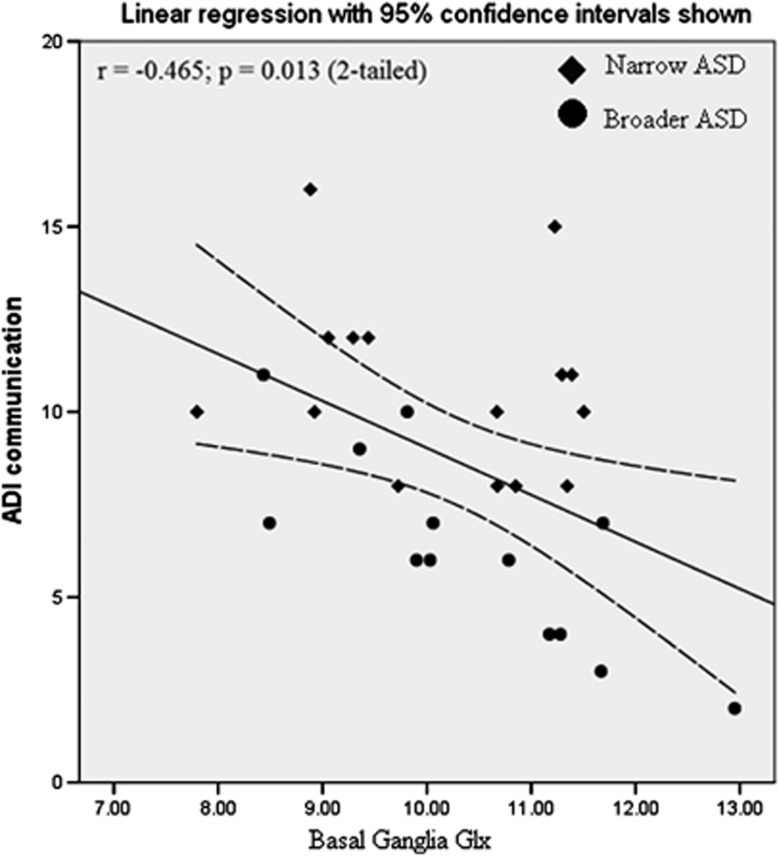
Association between basal ganglia glutamate and glutamine (Glx) and Autism Diagnostic Interview—Revised (ADI-R) Communication Subscale Score in individuals with autism spectrum disorder (ASD).

Across the combined ASD group (both broader and narrow phenotype), lower basal ganglia concentration of Glx was significantly correlated with worse scores on the ADI-R Communication Scale (i.e. more abnormal Glx concentrations were associated with greater communication impairment *(r*=−0.465, *P*=0.013, *n*=28; see Figure 4). This correlation was specific to this metabolite and this symptom domain: no correlations were seen in other metabolites or domains.

We further examined this association within each group separately. There was no significant correlation in the narrowly defined autism group (*r*=−0.224, *P*=0.422), but there was in those with the broader phenotype (*r*=−0.805, *P*=0.001).

## Discussion

We found that adults with ASD have a significantly reduced Glx concentration in the basal ganglia as compared with controls. Our preliminary evidence further suggests that that this reduction was (1) regionally specific, that is, there were no significant differences in the other cortical regions we examined; (2) a potential final common pathway in ASD, as it was present in both ‘narrow' and ‘broadly' defined ASD; and (3) was associated with some aspects of clinical variation (social communication).

We suggest that it is unlikely that our findings can be fully explained by potential confounds, such as differences in voxel tissue composition, age, IQ or medication. There were no significant between-group differences in voxel grey matter, white matter and CSF, and all metabolite values were corrected for CSF %. Demographic variables such as age, gender and IQ were not different between the groups, all participants were right handed and all of the individuals we studied were psychotropic medication naive according to self-report.

This is the first [^1^H]MRS study to report on Glx in the basal ganglia of adults with ASD. Our finding of reduced Glx in the basal ganglia (predominantly the lentiform nuclei) agrees with studies finding reductions of Glx in the cingulate cortex and the thalamus,^19^ but contrasts with our previous finding of *increased* Glx in the amygdala-hippocampal cortex in adults with ASD.^18^ Also, similar to prior studies, we found no differences in the parietal cortex.^18^

Taken together, these results demonstrate that, rather than being a ‘global' neurobiological abnormality, Glx changes seen in ASD are highly regionally specific, suggesting that the underlying neurobiological cause(s) are also localized. Reductions in Glx could result simply from a local reduced density of glutamatergic synapses and neurons, such as reduced storage capacity and turnover, but could also be the product of alterations in glutamate and glutamine metabolism.

In neurons, glutamate is synthesized from glutamine via glutaminase, but after release in the synapse, glutamate is converted back into glutamine in glial cells, by glutamine synthetase. Glutamate is also converted to GABA by the neuronal enzyme glutamate decarboxylase (GAD).^5^

Alterations in GAD expression could be a potential explanation for the fact that we observed reduced Glx in the basal ganglia. This hypothesis would also help to reconcile these results with the suggestion that individuals with ASD have an *inhibitory* signalling deficit and an *increased* ratio of excitatory glutamate to inhibitory GABA transmission.^8, 31^ It is possible that, while the Glx signal was reduced, the *ratio* between glutamate and GABA was still increased. Reduced glutamate would be expected to lead to a corresponding reduction in GABA synthesis, as glutamate is the precursor of GABA. If GAD activity were reduced, one would expect a lower GABA:Glx ratio.

Unfortunately, there have not yet been any studies examining GAD expression in the basal ganglia in ASD, but studies of other brain regions have shown regionally specific differences associated with the disorder. For example, GAD has been reported to be *decreased* in cerebellar Purkinje cells,^32^ but increased in cerebellar interneurons,^33^ in ASD.

A further possibility is that the observed differences in Glx are secondary to alterations in other neurotransmitter systems. For example, the basal ganglia are densely innervated by serotonergic projections, which exert complex modulatory effects on glutamate and GABA release.^34^ We and others reported reduced density of cortical 5HT2A (serotonin 2A) receptors and serotonin transporter in the cortex and midbrain,^35, 36, 37^ (although see Girgis *et al.*^38^) the same pattern has been found in the parents of children with ASD.^39^ Further, some have reported that children with ASD have significant differences in serotonin synthesis.^40^ Hence, it could be that serotonergic abnormalities underlie the differences in Glx we observed—either indirectly via influences on neurodevelopment or through direct action on glutamate metabolism.

Also, in the context of previous findings, our results suggest that within ASD age may be an important moderator of both cortical and subcortical differences in brain Glx. Specifically, prior [^1^H]MRS studies in *children* with ASD reported widespread decreases in cortical Glx^14^—but no differences in the basal ganglia.^16, 17^ This is the opposite of the pattern we observed in adults, namely no differences in the cortical regions (DLPFC and parietal lobe), but a reduction in the basal ganglia. This is consistent with the idea of autism as a disorder of brain maturation.^41^ We were unable to address age effects directly, as this study did not include children—but this is a focus of our ongoing studies.

The correlation between basal ganglia (Glx) and the severity of social communication impairments in ASD is consistent with the known involvement of this area in various aspects of language and communication. For example, functional imaging studies demonstrated that the caudate and putamen are involved in ‘higher level' aspects of language, such as inferring with the implied as opposed to literal meaning of sentences,^42^ and resolving ambiguous sentences—functions that are characteristically impaired in ASD. This correlation was specific to the basal ganglia, however, with no significant correlation seen in the DLPFC, an area also known to be involved in communication and Theory of Mind.^28^ One explanation for this could be that alterations in neurotransmitters other than glutamate are responsible for DLPFC dysfunction in autism; serotonin is one possibility, as a previous study found that lower cortical 5HT2A receptor binding is associated with communication impairments in ASD.^37^

We are only able to report a correlation between ASD in adults (and in particular, Social Communication symptoms as measured by the ADI-R) and reduced basal ganglia Glx levels. Hence, we cannot be certain whether the differences in Glx are the *cause* of the ASD symptoms. It is possible that they represent a downstream *effect* of the symptoms if, for example, the lack of social interaction or high-level language use engaged in by people with ASD led, over time, to neuroplastic changes in corticobasal circuits. However, we do not believe that this can explain all our results, because the ADI is a measure childhood (developmental) symptoms in the first years of life.

In addition to the findings related to glutamate, discussed above, we also observed reduced levels of Cho, Cr and NAA in the basal ganglia, and of Cr and NAA in the DLPFC, in individuals with ASD, although only the basal ganglia Cr difference survived conservative Bonferroni correction. The finding of reduced Cr and NAA in both the basal ganglia and in the DLPFC is consistent with previous [^1^H]MRS studies in this area in ASD.^14, 15, 22^ However, we have previously^29^ reported *increased* NAA, Cr and Cho in the *medial* prefrontal cortex of adults with Asperger's syndrome, underlining that the differences seen are regionally specific. Also in line with prior work,^18, 29^ we did not observe any significant metabolite differences in the parietal cortex control region. This confirms that the effects observed in the basal ganglia and the DLPFC are regionally specific.

As both Cr and NAA are involved in neuronal energy metabolism, our finding of local reductions in these metabolites in the basal ganglia and the DLPFC may indicate either metabolic dysfunction in these areas or a reduced density of metabolically active neurons. If the latter, this may also explain the observed reduction in Cho in the basal ganglia (as this is a component of cell membranes).

Another important implication of our finding of reduced Cr in ASD in the basal ganglia and DLPFC is that it may be invalid to express the concentration of metabolites such as NAA and Glx as ratios to Cr in the same voxel. This approach is commonly used in [^1^H]MRS studies,^22, 43^ as it is widely assumed that Cr is constant; our findings suggest the this is not true in adults with ASD.

However, our study does have a number of limitations. We obtained [^1^H]MRS data on a 1.5 T MRI scanner. At 1.5 T, it is not possible to distinguish between the compounds that contribute to the ‘Glx' signal, that is, glutamate and glutamine. Future studies at 3 T or higher are needed to distinguish these compounds, but previous studies have cautiously attributed reductions in Glx to glutamate, as glutamate constitutes the most abundant central neurotransmitter.^14, 15, 18^

Another limitation is that we only recruited people with a normal or above-normal IQ, and excluded those with a history of epilepsy or seizures. This served to increase the homogeneity of the sample and ensure that any differences observed were associated with symptoms of ASD *per se*, but it means that the results may not be representative of the entire ASD population, as many people with ASD also suffer from a below-normal IQ and/or epilepsy.^1^ Future studies should examine this population.

In summary, we found preliminary evidence that adults with ASD (both narrowly and broadly defined) have significant differences in brain glutamate and/or glutamine metabolism. This may be a final ‘common pathway' in the disorder, and underpin some clinical symptoms. Further work is required to determine the cause(s) of this putative abnormality.

## Figures and Tables

**Table 1:** Participant demographic and clinical characteristics

	*Narrow ASD*	*Broader ASD*	*Control*	*F*	P*-value*
Number	15	13	14	N/A	
Number female	1	1	3		
Age (years)	29 (6.0)	27 (6.4)	34 (8.8)	2.86	0.07
FSIQ	95 (13)	103 (16)	107 (21)	1.63	0.21
VIQ	95 (16)	101 (20)	106 (19.0)	1.21	0.31
PIQ	96 (19)	106 (12)	107 (21)	1.47	0.24
ADI-R A domain	16.5 (4.2)	8.7 (2.4)	N/A		
ADI-R B domain	10.7 (2.4)	6.3 (2.6)			
ADI-R C domain	3.9 (0.8)	1.8 (1.5)			

Abbreviations: ADI-R, Autism Diagnostic Interview—Revised; Domain A, social interaction; FSIQ, full-scale IQ; Domain B, communication; Domain C, restricted and repetitive patterns of behaviour; HFA, high functioning autism; N/A, not applicable; PIQ, performance IQ; VIQ, verbal IQ.

Values are expressed as mean (s.d.), unless otherwise indicated.

*Note*: The healthy control group did not receive an ADI-R assessment. Therefore, there are no ADI-R scores for this group.

**Table 2:** Voxel tissue composition of grey matter, white matter and CSF

								*ANOVA*	
*Region*	*Measure*	*Narrow ASD*	*s.d.*		*s.d.*	*Control*	*s.d.*	*F*	P*-value*
Basal ganglia	Grey matter	62.20%	12.94%	64.99%	6.01%	54.31%	16.49%	2.65	0.084
	White matter	35.68%	13.07%	33.04%	5.12%	43.84%	17.13%	2.73	0.078
	CSF	2.09%	1.45%	1.92%	1.57%	1.82%	1.66%	0.16	0.849
									
DLPFC	Grey matter	45.35%	14.48%	45.32%	14.01%	2.17%	14.37%	0.38	0.685
	White matter	48.79%	17.57%	49.37%	16.70%	54.06%	17.34%	0.68	0.685
	CSF	5.67%	4.03%	5.07%	3.39%	4.03%	3.58%	0.76	0.476
									
Parietal lobe	Grey matter	44.47%	7.94%	47.61%	7.62%	44.89%	6.33%	1.03	0.366
	White matter	35.16%	14.10%	34.47%	11.98%	38.39%	11.43%	0.35	0.706
	CSF	19.34%	14.28%	15.75%	6.86%	16.09%	7.56%	0.48	0.625

Abbreviations: ANOVA, analysis of variance; ASD, autism spectrum disorder; CSF, cerebrospinal fluid; DLPFC, dorsolateral prefrontal cortex.

**Table 3:** Metabolite concentrations in BG, DLPFC and parietal cortex [^1^H]MRS voxels and group differences

					*ANOVA*		Post hoc t*-tests*—P*-values*^b^		
*Region*	*Metabolite*^a^	*Narrow ASD*	*Broader ASD*	*Control*	*F*	P*-values*	*Narrow ASD* vs *controls*	*Broad ASD* vs *controls*	*Broad ASD* vs *narrow ASD*
Basal ganglia	Glx	10.12 (1.14)	10.43 (1.26)	12.34 (1.45)	12.63	**<0.0001**^c^	<0.001	0.001	0.25
	Cho	1.205 (0.19)	1.098 (0.14)	1.324 (0.19)	5.83	0.006^d^	0.212	0.004	0.44
	Cr	5.097 (0.65)	5.323 (0.53)	6.435 (1.25)	10.02	**0.0003**^c^	<0.001	0.004	0.47
	NAA	5.454 (0.81)	5.486 (0.42)	6.458 (1.52)	4.54	0.017^d^	0.030	0.045	0.32
									
DLPFC	Glx	7.492 (1.11)	7.156 (1.51)	8.007 (1.42)	1.36	0.27	—	—	0.32
	Cho	1.129 (0.21)	1.110 (0.16)	1.271 (0.27)	2.35	0.11	—	—	0.93
	Cr	4.074 (0.48)	4.021 (0.49)	4.602 (0.60)	5.32	0.009^d^	0.027	0.017	0.64
	NAA	5.954 (0.79)	5.955 (0.47)	6.660 (0.95)	4.07	0.025^d^	0.047	0.057	0.22
									
Parietal	Glx	10.607 (2.79)	9.790 (1.65)	11.028 (1.62)	1.31	0.28	—	—	0.41
	Cho	1.112 (0.37)	1.048 (0.13)	1.149 (0.25)	0.53	0.59	—	—	0.524
	Cr	5.461 (1.42)	5.140 (0.81)	5.382 (0.70)	0.39	0.68	—	—	0.19
	NAA	7.998 (2.32)	7.472 (0.76)	7.887 (1.06)	0.48	0.62	—	—	0.31

Abbreviations: ANOVA, analysis of variance; ASD, autism spectrum disorder; BG, basal ganglia; Cho, choline; Cr, creatine; DLPFC, dorsolateral prefrontal cortex; Glx, glutamate and glutamine; [^1^H]MRS, proton magnetic resonance spectroscopy; NAA, *N*-acetylaspartate.

a Data are expressed as mean (s.d.).

b Post hoc *t*-tests comparing ASD cases with controls were only performed when ANOVA was significant at uncorrected *P*<0.05.

c Bold figures show values significant at *P*=0.05 level, with Bonferroni correction over 12 comparisons, that is, uncorrected *P*<0.0047.

d Significant at *P*=0.05 level before Bonferroni correction.
